# Randomized trial comparing daily interruption of sedation and nursing-implemented sedation algorithm in medical intensive care unit patients

**DOI:** 10.1186/cc6908

**Published:** 2008-05-20

**Authors:** Marjolein de Wit, Chris Gennings, Wendy I Jenvey, Scott K Epstein

**Affiliations:** 1 Pulmonary and Critical Care Division, Department of Internal Medicine, School of Medicine, Virginia Commonwealth University, PO Box 980050, Richmond, VA 23298-0050, USA; 2 Department of Biostatistics, School of Medicine, Virginia Commonwealth University, Box 980032, Richmond, VA, USA; 3 Tufts University School of Medicine, 145 Harrison Street, Boston, MA 02111, USA

## Abstract

**Introduction:**

Daily interruption of sedation (DIS) and sedation algorithms (SAs) have been shown to decrease mechanical ventilation (MV) duration. We conducted a randomized study comparing these strategies.

**Methods:**

Mechanically ventilated adults 18 years old or older in the medical intensive care unit (ICU) were randomly assigned to DIS or SA. Exclusion criteria were severe neurocognitive dysfunction, administration of neuromuscular blockers, and tracheostomy. Study endpoints were total MV duration and 28-day ventilator-free survival.

**Results:**

The study was terminated prematurely after 74 patients were enrolled (DIS 36 and SA 38). The two groups had similar age, gender, racial distribution, Acute Physiology and Chronic Health Evaluation II score, and reason for MV. The Data Safety Monitoring Board convened after DIS patients were found to have higher hospital mortality; however, no causal connection between DIS and increased mortality was identified. Interim analysis demonstrated a significant difference in primary endpoint, and study termination was recommended. The DIS group had longer total duration of MV (median 6.7 versus 3.9 days; *P *= 0.0003), slower improvement of Sequential Organ Failure Assessment over time (0.70 versus 0.23 units per day; *P *= 0.025), longer ICU length of stay (15 versus 8 days; *P *< 0.0001), and longer hospital length of stay (23 versus 12 days; *P *= 0.01).

**Conclusion:**

In our cohort of patients, the use of SA was associated with reduced duration of MV and lengths of stay compared with DIS. Based on these results, DIS may not be appropriate in all mechanically ventilated patients.

**Trial registration:**

ClinicalTrials.gov NCT00205517.

## Introduction

The method of sedation administration has been shown to impact duration of mechanical ventilation (MV) in critically ill patients. Daily interruption of sedation (DIS) has been shown to decrease duration of MV [1,2]. While some studies examining the effectiveness of sedation algorithms (SAs) have found a decrease in duration of MV by 2.6 to 5.9 days, others have found no difference, although these latter trials were not randomized [3-6]. Additionally, DIS appears to be protective against the development of further organ dysfunction, while studies examining SA found neither a worsening nor an improvement of organ dysfunction [3,4,7]. Both DIS and SA have been associated with decreased administration of sedatives and opioids [1,4].

Studies examining DIS and SA have compared these strategies to conventional care or modifications of the initial strategy [8]. In the recently published Awakening and Breathing Controlled (ABC) trial, both groups were managed with a spontaneous breathing trial (SBT) and one group was also managed with a modified DIS protocol in which analgesics could be continued if deemed necessary for pain [2]. In both arms of the study, patients could be managed by an SA, but this was not mandated. To our knowledge, no study has directly compared DIS with SA. We therefore designed a randomized study comparing DIS and SA.

## Materials and methods

The study was conducted in accordance with the ethical standards of the Virginia Commonwealth University Institutional Review Board (IRB) and the Declaration of Helsinki, and the study was performed at Virginia Commonwealth University Medical Center (Richmond, VA, USA). The IRB approved the study, and written consent was obtained. Adults 18 years old or older receiving invasive MV in the closed medical intensive care unit (ICU) were eligible for study participation unless they met exclusion criteria. Exclusion criteria were neuromuscular blockade, severe chronic neurocognitive dysfunction requiring assistance with most activities of daily living, transfer from another ICU, tracheostomy at the time of study enrollment, or inability to obtain consent before the time point when sedation was to be interrupted.

**Figure 1:**
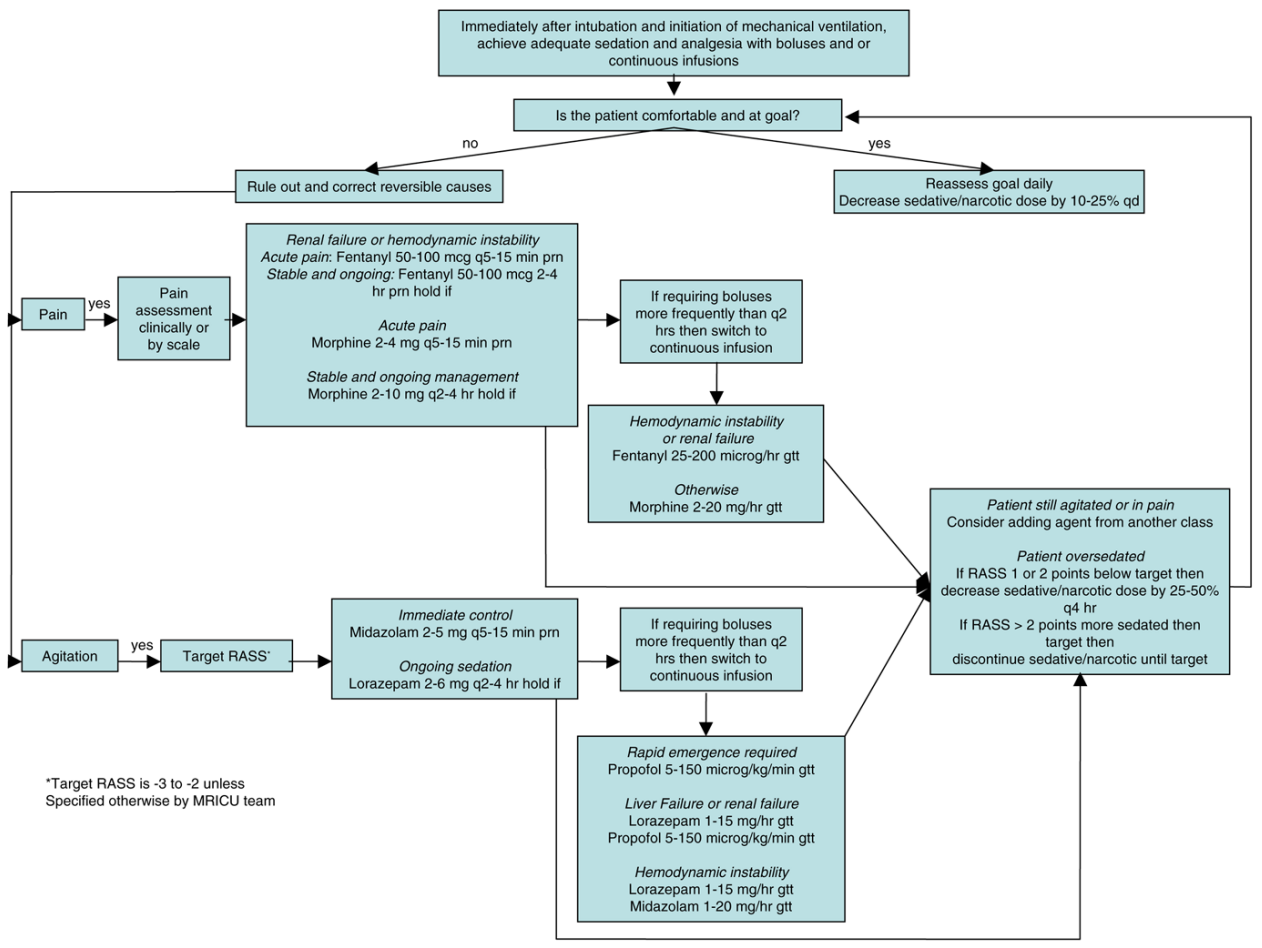
The sedation algorithm used in this study. gtt, drop; MRICU, medical respiratory intensive care unit; prn, as necessary (*pro re nata*); q, every; qd, each day (*quaque die*); RASS, Richmond Agitation-Sedation Scale.

Patients were randomly assigned to one of two sedation strategies: DIS or nursing-implemented SA. Sedation in all patients was managed according to the algorithm up to the time of randomization. The SA was developed locally by the medical ICU physicians, pharmacist, and nursing staff and was based on the algorithm developed by Brook and colleagues [3] and on Society of Critical Care Medicine guidelines [9]. The algorithm goals were to maximize the use of boluses, minimize the duration of continuous intravenous infusion of sedation, and treat pain with opioids (Figure 1). It included daily attempts to decrease sedation dosages. The Richmond Agitation-Sedation Scale (RASS) was used to measure sedation level, and sedation was titrated to a goal RASS score of -2 to -3 unless otherwise specified by the ICU team (Table 1) [10]. The ICU nursing staff underwent a 2-month introductory period in the use of the algorithm. After educational training, the algorithm underwent a 1-month run-in period prior to study initiation. During the trial, nurses assigned to study patients were asked daily whether they required any clarification regarding the algorithm. Nurses administered sedatives and opiates as mandated by the algorithm. Study investigators monitored compliance with the algorithm by evaluating sedation levels in patients randomly assigned to SA on two occasions per day, with the timing of the events being at the discretion of investigators and separated by at least 2 hours.

**Table 1:** Richmond Agitation-Sedation Scale

Score	Term	Description
+4	Combative	Overtly combative or violent and an immediate danger to staff
+3	Very agitated	Pulls on or removes tube(s) or catheter(s) or has aggressive behavior toward staff
+2	Agitated	Frequent nonpurposeful movement or patient ventilator dyssynchrony
+1	Restless	Anxious or apprehensive but movements not aggressive or vigorous
0	Alert and calm	
-1	Drowsy	Not fully alert but has sustained (> 10 seconds) awakenings, with eye contact, to voice
-2	Light sedation	Briefly (< 10 seconds) awakens with eye contact to voice
-3	Moderate sedation	Any movement (but no eye contact) to voice
-4	Deep sedation	No response to voice, but any movement to physical stimuli
-5	Unarousable	No response to voice or physical stimulation

DIS was performed initially would leave initially out as outlined by Kress and colleagues [1]. Forty-eight hours after initiation of MV, all sedatives and opioids administered as either continuous infusions or bolus infusions were discontinued until the patient was awake or agitated. Sedation was typically interrupted in the morning, but timing was based on practicalities such as daily rounds, procedures, and travel outside the ICU. Patients were observed continuously by a study investigator (MdW or WIJ) during sedative and opioid interruption. Awake was defined as being able to perform at least three of the following four commands: (a) open eyes, (b) visually track the investigator, (c) stick out tongue, and (d) squeeze hand [1]. Agitation was defined *a priori *as an RASS score of greater than 0. Study investigators decided if and when to resume sedation. Patients randomly assigned to DIS were not managed with the SA, and all sedation management was left to the discretion of the ICU team. However, clinicians were instructed to target an RASS score of -2 to -3 unless the ICU team felt that a different sedation depth was necessary. Clinicians titrated sedatives and opioids throughout the day when study investigators were not present. Patients treated by DIS had sedation levels recorded at the time sedation was interrupted and again when sedation was resumed. Clinicians involved in the care of study patients did not initiate sedation interruption, nor were they involved in the decision to resume sedation.

After the third patient randomly assigned to DIS experienced a study-related adverse event, the DIS protocol was amended because of safety concerns. During sedation interruption, the patient developed hypertension and subsequent tachycardia, tachypnea, and patient-ventilator asynchrony. Treatment with sedatives led to resolution of the hypertension and tachycardia, but the tachypnea and patient-ventilator asynchrony persisted and necessitated the administration of a neuromuscular-blocking agent. The protocol was amended to resume sedation if any of the following vital sign changes occurred: tachypnea of greater than 35 breaths per minute for more than 5 minutes, arterial oxygen saturation of less than 90%, heart rate of greater than 140 beats per minute or sustained change of greater than 20% in either direction, or systolic blood pressure of greater than 180 mm Hg or less than 90 mm Hg. These changes were based on a study evaluating the efficacy of SBTs [11]. Once a patient was awake, agitated, or developed vital sign changes, sedation was resumed at half the previous dose. Boluses could be administered if deemed necessary to treat agitation or vital sign changes. MV weaning was standardized in both groups through the use of daily SBTs [11].

### Data analysis

The purpose of the study was to compare the time to a successful extubation in the two groups. The time to successful extubation was compared using a Kaplan-Meier survival analysis. The subjects in the two randomly assigned groups were analyzed using the intent-to-treat principle. Statistical significance was determined using a log-rank test. This statistical test allowed for the fact that not all patients achieved a successful extubation. That is, all subjects were included in the analysis even if they did not have a successful extubation. Unsuccessful extubations created censored observations. Censoring occurred if a patient died on MV, withdrew from the study, required reintubation (within 72 hours of extubation), or underwent tracheostomy. We chose to censor for reintubation and tracheostomy because these events have been shown to be affected by sedation method.

We also computed 28-day ventilator-free survival, which is defined as the number of days within the first 28 days after enrollment during which the patient was alive and breathing without assistance, if the period of unassisted breathing was 72 hours or longer [12]. Finally, we computed the total duration of MV, which was calculated as the time from randomization until extubation. This included time after reintubation, tracheostomy, or MV up to 28 days after enrollment [13].

One interim analysis was scheduled when half the targeted number of patients were enrolled. The trial was set to be halted at interim analysis if the *P *value was less than alpha and alpha = 0.001. For the remainder of the analyses, alpha was set to 0.05. However, after a patient experienced complications during sedation interruption, the Data Safety Monitoring Board (DSMB) chose to closely follow all reported adverse events and perform an interim analysis as necessary based on clinical expertise and safety concerns. Investigators submitted all predefined adverse events to the DSMB within 2 business days; these events were unplanned extubation, reintubation, hospital mortality, and an RASS score of greater than 2. The DSMB performed one interim analysis when investigators noted that patients randomly assigned to DIS had higher mortality.

Sedation levels were compared using mixed-model repeated measures analysis of variance (ANOVA; for RASS variable) or a generalized estimating equation (for the awake variable) model [14]. The total dose of sedatives and opioids was recorded and converted to midazolam and fentanyl equivalents using referenced conversion formulas [15,16]. Medication doses were compared using the Wilcoxon rank sum test. A mixed-effects ANOVA was used to model severity of illness using the Sequential Organ Failure Assessment (SOFA) over time [17]. Hospital and ICU lengths of stay were compared by log-rank with censoring for study withdrawal. Other variables collected included age, race, severity of illness as measured by Acute Physiology and Chronic Health Evaluation II (APACHE II), and MV reason [18]. Normally distributed data are reported as mean and 95% confidence interval (CI). Non-normally distributed data are reported as median and interquartile range or as median and 95% CI.

### Power calculation

Brook and colleagues [3] observed a median time on MV to be 2.3 days in the SA group compared with 4.9 days for the control group. Kress and colleagues [1] observed median times of 4.9 days in the DIS group and 7.3 days for the control group. *A priori*, a meaningful difference between the two groups was set at 2 days. With a censoring rate of 13%, 268 patients were deemed necessary to detect a significant difference between groups using a log-rank test with 80% power and a two-sided test.

## Results

Seventy-five patients were enrolled. One patient withdrew immediately after randomization (DIS group). Baseline characteristics of 74 patients revealed no difference in age, gender distribution, racial composition, severity of illness, and reason for MV (Table 2). At study entry, DIS and SA patients had similar RASS scores, were equally likely to be awake, and had received similar doses of sedatives and opioids. Interim analysis was performed early because of safety concerns and revealed increased hospital mortality in patients treated by DIS. Because the study primary endpoint might affect mortality, the DSMB reviewed this. The study was designed *a priori *to detect a 2-day difference in MV duration, and the DSMB recommended study termination after this endpoint was reached. It should be noted that investigators were not members of the DSMB and were not involved in the DSMB's data analysis. The study was not terminated because of the finding of increased hospital mortality in patients treated by DIS.

**Table 2:** Baseline characteristics for patients randomly assigned to daily interruption of sedation (DIS) and sedation algorithm (SA)

	DIS *n *= 36	SA *n *= 38	*P *value
Age in years, mean (95% CI)	52 (47.4, 56.5)	51 (46.8, 55.8)	0.84
Gender, female	19	20	0.99
Race, African-American/white/other	18/17/1	20/17/1	0.97
Reason for mechanical ventilation			0.52
Pneumonia/Acute lung injury	17	14	
Sepsis	6	5	
Delirium/neurologic	5	6	
Cardiac	2	6	
Asthma/Chronic obstructive pulmonary disease	1	2	
Other	5	5	
APACHE II score	26 (22.9, 28.8)	24 (21.6, 27.4)	0.52
Sequential Organ Failure Assessment score	10 (8.2, 10.9)	9 (7.6, 10.3)	0.50
Midazolam equivalents before randomization in mg/kg, median (IQR)	0.5 (0.05, 2.61)	0.6 (0.0, 3.73)	0.81
Fentanyl equivalents before randomization in μg/kg, median (IQR)	0.4 (0.0, 2.93)	0.8 (0.0, 2.27)	0.52
Propofol before randomization in μg/kg, median (IQR)	90 (0, 29,625)	0 (0, 49,956)	0.79
Awake, number	13	14	0.95
Richmond Agitation-Sedation Scale score, mean (95% CI)	-3 (-3.4, -2.3)	-3 (-3.0, -2.0)	0.37

APACHE II, Acute Physiology and Chronic Health Evaluation II; CI, confidence interval; IQR, interquartile range.

### Censoring

Thirty-six patients were censored. Patients randomly assigned to DIS were significantly more likely to have censored observations (DIS 24 versus SA 12; *P *= 0.004). Reasons for censoring included reintubation (DIS 8 and SA 5), death on MV or medical treatment withdrawn (DIS 8 and SA 6), tracheostomy (DIS 2 and SA 0), and study withdrawal (DIS 6 and SA 1). Patients randomly assigned to DIS were more likely to withdraw from the study (*P *= 0.03). Five patients (4 DIS and 1 SA) were withdrawn at the request of the legally authorized representative because of concerns that patients were insufficiently sedated. Two patients randomly assigned to DIS were withdrawn at the request of the attending physician because the patients were not felt to be appropriate candidates for DIS. (The first patient was the subject who developed the adverse events described above, and the second subject was a patient with acute fulminant liver failure with a concern for increased intracranial pressure.)

### Mechanical ventilation and length of stay

**Figure 2:**
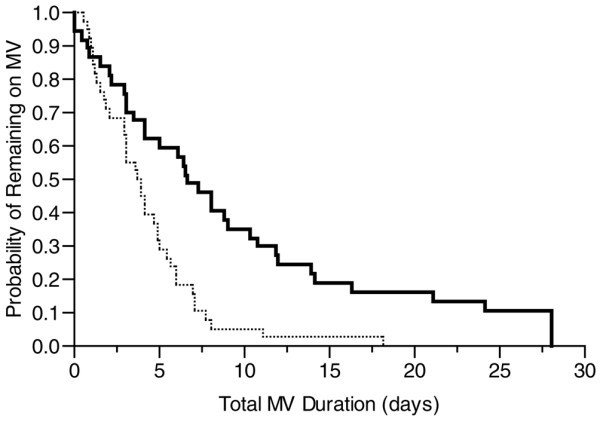
Kaplan-Meier survival curve of total duration of mechanical ventilation (MV) for patients treated by daily interruption of sedation (thick line) and sedation algorithm (thin line) (*P *= 0.0003).

The time to successful extubation from MV was 4.0 days longer in the DIS group (median 8.1 days, 95% CI 4.1, undeterminable days for DIS versus 4.1 days, 95% CI 3.0, 4.9 days for SA). The total duration of MV was 2.8 days longer for the DIS group (Table 3). A Kaplan-Meier analysis graphing the total duration of MV shows that the probability of remaining on MV was reduced in the SA group (Figure 2). The 28-day ventilator-free survival was 7 days longer in the SA group compared with the DIS group (*P *= 0.004) (Table 3). Both the ICU and hospital lengths of stay were longer for DIS patients (Table 3).

**Table 3:** Comparison of outcome between daily interruption of sedation and sedation algorithm

	Daily interruption of sedation *n *= 36		Sedation algorithm *n *= 38		*P *value
	Median	95% CI	Median	95% CI	
Total duration of mechanical ventilation, days	6.7	4.1, 10.4	3.9	2.9, 4.9	0.0003
Intensive care unit length of stay, days	15	9.1, 21.2	8	6.5, 8.7	< 0.0001
Hospital length of stay, days	23	14.8, 28.7	12	11.3, 16.0	0.01
					
		
	Median	IQR	Median	IQR	
		
28-day ventilator-free survival	16.1	0.00, 21.77	23.1	19.16, 25.06	0.004
Midazolam equivalents, mg/kg-day	0.2	0.01, 1.48	0.4	0.01, 1.32	0.70
Fentanyl equivalents, μg/kg-day	0.5	0.09, 2.43	1.2	0.12, 2.44	0.36
Propofol, μg/kg-day	0	0.0, 5817.0	0	0.0, 6589.0	0.39

CI, confidence interval; IQR, interquartile range.

### Sedation

**Figure 3:**
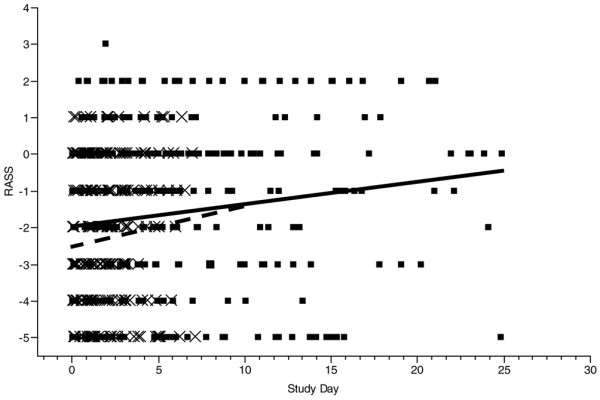
Mixed-model repeated measures comparison of Richmond Agitation-Sedation Scale (RASS) score over the course of mechanical ventilation in patients treated by daily interruption of sedation (solid line) and sedation algorithm (dashed line). The group treated by daily interruption of sedation had higher RASS scores (*P *= 0.049). Individual measurements are shown (× for sedation algorithm and ▪ for daily interruption of sedation).

A total of 671 RASS evaluations were performed (DIS 413 and SA 258). RASS score increased over time in both groups (*P *= 0.02), and the increases were similar for the two groups. However, DIS patients consistently had higher RASS values (0.6 units) than SA patients (Figure 3). The probability of being awake (as defined by Kress and colleagues [1]) did not change over time (*P *= 0.53), and there was no difference between the two groups (*P *= 0.78). Patients were awake on 46% of evaluations. Patients treated by DIS were agitated (RASS score > 0) during 18% of the evaluations, whereas patients treated by SA were agitated on 5% of the evaluations (*P *< 0.0001). There was no difference in the amount of sedatives and opioids administered between the two groups (Table 3).

### Sedation interruption

In the DIS group, assessments were made for sedation interruption on 173 occasions. In 92% of the cases, patients were receiving continuous infusions; in 4% of the cases, patients were receiving boluses; in the remaining 4% of the cases, patients were not receiving sedation as it had been held from the previous day. On 94 occasions, patients were either awake (as defined by Kress and colleagues [1] on 89 occasions) or agitated (RASS score > 0 on 5 occasions) and did not require sedation interruption. On the 79 occasions when sedation was interrupted, sedation was interrupted for a mean of 3.5 hours (95% CI 2.61, 4.37 hours). Patients subsequently met the definition of awake on 22 occasions (12 occasions without agitation or vital sign changes and 10 occasions with both agitation and vital sign changes). On 47 occasions, patients developed agitation and were not awake; on 25 occasions, patients developed vital sign changes (in 8 out of 33 patients who were enrolled in the modified protocol). Extreme tachypnea at a mean rate of 51 breaths per minute as measured on the ventilator (95% CI 45.0, 57.3 breaths per minute) was the reason for sedation resumption in 22 out of 25 occasions.

### Severity of illness

**Figure 4:**
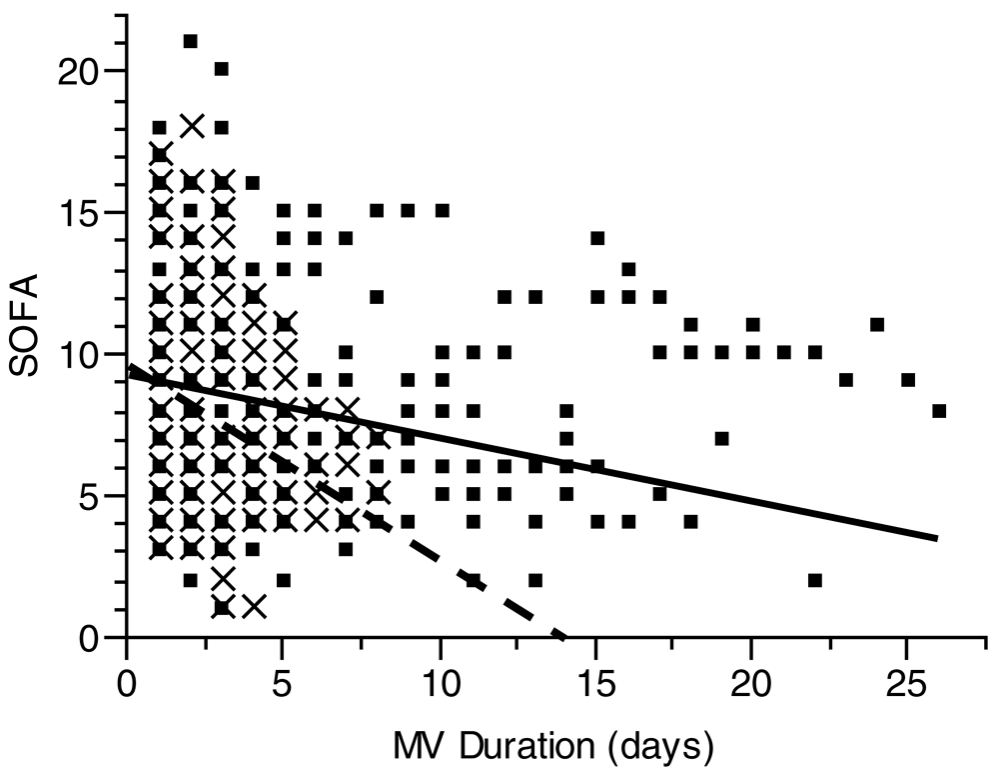
Sequential Organ Failure Assessment (SOFA) over time for the group of patients treated by daily interruption of sedation and sedation algorithm. The individual scores are represented for each group (▪ for daily interruption of sedation and × for sedation algorithm); the lines represent the composite SOFA for each group (thick line for daily interruption of sedation; dashed line for sedation algorithm). The SOFA improved more rapidly for the sedation algorithm group (*P *= 0.025). MV, mechanical ventilation.

SOFA decreased throughout the study in both groups (*P *< 0.001). However, the SA group had a more rapid improvement in SOFA: 0.70 units per day in the SA group compared with 0.23 units per day in the DIS group (*P *= 0.025) (Figure 4).

### Mortality

Thirteen patients treated by DIS died in hospital compared with 7 treated by SA (*P *= 0.04, analysis excluded patients who withdrew from the study). Eight patients treated by DIS died in the ICU compared with 5 treated by SA (*P *= 0.20, excluding participants who withdrew). The DSMB reviewed the causes of mortality and could not determine a common etiology. In particular, 8 patients randomly assigned to DIS who died had progressive deterioration and subsequently had care withdrawn at the recommendation of the medical team. Three of 5 patients randomly assigned to SA who died had care withdrawn; in 2 cases, this was at the recommendation of the medical team and in 1 case at the request of the patient's next of kin.

## Discussion

The main findings from our study are the following: compared with DIS, the use of an SA was associated with (a) decreased duration of MV, (b) more rapid resolution of critical illness, and (c) shorter ICU and hospital lengths of stay. (d) Additionally, DIS was associated with less deep sedation levels but was not associated with the administration of less sedative medication compared with the use of SA.

Early in the study, a patient developed serious study-related adverse events. When an interim analysis demonstrated increased mortality rates in the DIS group, the DSMB, guided by safety concerns, examined the primary endpoint as this could potentially explain the mortality findings. When it was noted that the *a priori*-defined difference in time to successful extubation across the two groups exceeded 2 days, the DSMB recommended study termination. It should be noted that the study was not terminated because of the mortality findings.

More patients treated by DIS were withdrawn from the study. After study withdrawal, these patients were treated using the ICU SA. This could have resulted in eliminating differences in total MV duration between the two groups. However, the DIS group had a longer total MV duration by 2.8 days (*P *= 0.0003), suggesting a true effect of DIS on MV outcome. Ventilator-free survival was also improved by 7 days in the SA group, although this did not reach significance (*P *= 0.004, which is greater than the preset alpha of 0.001 for interim analysis).

DIS has been advocated by the Surviving Sepsis Campaign [19] and the Institute for Healthcare Improvement [20] as a tool to improve patient outcome. Kress and colleagues [21] previously demonstrated that use of DIS shortens the duration of MV, decreases sedative administration, increases frequency of wakefulness from 9% to 86%, and decreases the frequency of neurodiagnostic testing. The recent ABC trial also found that sedation interruption, when combined with an SBT, resulted in improved ventilator-free survival [2]. Our study differs significantly from that of Kress and colleagues [21] and the ABC trial in a number of ways. First, we compared DIS with a carefully implemented nurse-driven SA rather than with a non-algorithmic approach. Although some patients in the ABC trial may have been treated by an algorithm, this was not a requirement. Second, the majority of our patients were women and half of patients were African-American. Racial and gender differences in medication metabolism are well described, and studies have found that patient characteristics influence medication benefit [22-25]. Third, in the ABC trial, clinicians could continue the use of analgesics if they felt this was necessary, and this occurred in 15% of patients whose sedatives were discontinued [2]. Additionally, if patients required escalating doses of sedatives, they were not considered candidates for sedation interruption. Our protocol was modeled after that of Kress and colleagues [21]. In our protocol, escalating doses of sedatives did not preclude the discontinuation of sedative and opioid medications unless a patient was agitated at the time the medications were due to be interrupted.

Although DIS patients had higher RASS scores, they did not receive less sedation than the comparison group, an observation that may result from several factors. It is conceivable that the SA used in our study resulted in more medication administration than the control group in the study of Kress and colleagues. Alternatively, our DIS group may have received more sedation because of the protocol modification requiring sedation resumption because of changes in vital signs. Indeed, in a third of occasions, sedation was resumed because of vital sign criteria. Finally, it is also possible that the higher rates of agitation in the DIS group resulted in an increased need for larger doses of sedation, thereby eliminating dosage differences between the two groups.

Another possibility is that our patient population contained a high proportion of patients with alcohol and other drug use disorders. In Richmond, the prevalence of these disorders is 18%, approximately twice the national rate, and our institution cares for many patients with these disorders [26,27]. In a retrospective study, we found that 39% of our mechanically ventilated critically ill patients have alcohol and other drug use disorders, and this rate likely under-represents the true rate due to the retrospective nature of the study [28]. Additionally, alcohol and other drug use disorders typically are coexisting diagnoses in our patients and are not usually the primary reason for requiring MV. The prevalence of alcohol and other drug use disorders in our medical ICU patients is likely to be substantially higher than in the study of Kress and colleagues and the ABC trial. Neither of these two studies reported rates of coexisting alcohol and other drug use disorders. Although one patient in the study of Kress and colleagues required MV for a drug overdose, the ABC trial reported that only 1% of patients with alcohol withdrawal were enrolled in the study.

Alcohol withdrawal has been shown to be associated with longer duration of MV, and patients with alcohol use disorders can develop withdrawal syndromes if they are undersedated or have early withdrawal of sedation [29,30]. Additionally, sedative agents have been found to reduce the duration of alcohol withdrawal delirium, and opioids have been shown to decrease the stress response in critically ill patients with alcohol use disorders [31,32]. Patients with alcohol and other drug use disorders require a 2.5-fold increase in dosage administration of sedatives and a 5-fold increase in opioid dosage administration to achieve sedation levels similar to patients without these disorders [28]. It is possible that patients with alcohol and other drug use disorders may well be patients who require escalating doses of sedatives and opioids and who would not have had their sedation interrupted in the ABC trial. Additionally, it is possible that patients with these disorders did not have their analgesic medications discontinued in the ABC trial, thereby resulting in a blunted stress response during sedation interruption and minimizing the symptoms of withdrawal during sedation interruption [32]. The assessment of withdrawal syndromes rests principally on patient self-reporting of subjective sensations of irritability, nausea, headache, and tactile, visual, and auditory hallucinations. No objective criteria exist for assessing withdrawal in the non-verbal mechanically ventilated ICU patient [33]. We believe the hypertension, tachycardia, and tachypnea (that is, 'autonomic agitation') experienced by the third patient randomly assigned to DIS may be explained by withdrawal symptoms.

Based on the results of our study, DIS may not be the sedation strategy of choice in all mechanically ventilated patients. We cannot be sure that the sedative strategy alone was responsible for the difference in outcome as there are many uncontrolled factors (that is, comorbidities, severity of illness, organ failure, and so on) and temporally some patients died after leaving the ICU. However, our study raises some concerns about the applicability of DIS in all patients and highlights the need for additional randomized control trials. Previous trials examining DIS were done at institutions with expertise in sedation research and with a research coordinator at the bedside, which may limit generalizabiltity.

## Conclusion

In summary, in our cohort of patients, we found an SA to be superior to DIS in decreasing the duration of MV. DIS may not be effective in all patient populations and may potentially be harmful in some patient groups. This study raises the question of whether those with alcohol and other drug use disorders are one such population. Evaluation of sedation interruption in patients with alcohol and other drug use disorders requires further study.

## Key messages

• In our patient population, a nursing-implemented sedation algorithm was superior to a strategy of daily interruption of sedation in decreasing the duration of mechanical ventilation.

• Daily interruption of sedation was associated with less deep sedation but was not associated with dose reduction in sedative and opioid administration.

• In our patient population, a nursing-implemented sedation algorithm was associated with more rapid resolution of critical illness and shorter intensive care unit and hospital lengths of stay.

## Abbreviations

ABC = Awakening and Breathing Controlled; ANOVA = analysis of variance; APACHE = Acute Physiology and Chronic Health Evaluation, CI = confidence interval; DIS = daily interruption of sedation; DSMB = Data Safety Monitoring Board; ICU = intensive care unit; IQR = interquartile range, IRB = Institutional Review Board; MV = mechanical ventilation; RASS = Richmond Agitation-Sedation Scale; SA = sedation algorithm; SBT = spontaneous breathing trial; SOFA = Sequential Organ Failure Assessment.

## Competing interests

This study was funded by the National Institutes of Health (K23-GM-068842, M01-RR00065) and the American Lung Association (RT-053-N). The authors declare that they have no competing interests.

## Authors' contributions

MdW participated in study conception, study design, data acquisition, data analysis and interpretation, and drafting of the manuscript. CG participated in study design, data analysis and interpretation, and drafting of the manuscript. WIJ participated in data acquisition and drafting of the manuscript. SKE participated in study conception, study design, data analysis and interpretation, and drafting of the manuscript. All authors read and approved the final manuscript.
